# Knowledge, attitude and practice in managing patients with asymptomatic hyperuricaemia among primary care doctors in Malaysia: A cross-sectional study

**DOI:** 10.51866/oa.970

**Published:** 2025-10-30

**Authors:** Mohamad Faris Rusydi Rusly, Mazapuspavina Md. Yasin, Mariam Mohamad, Khairatul Nainey Kamaruddin

**Affiliations:** 1 MB BCH BAO, MMed Fam Med, Department of Primary Care Medicine, Faculty of Medicine Universiti Teknologi MARA, Jalan Hospital, Sungai Buloh, Selangor, Malaysia. Email: khairatul@uitm.edu.my; 2 MBBS, MMed Fam Med, Klinik Kesihatan Kampar (Ministry of Health Malaysia), Jalan Degong, Kampar, Perak, Malaysia.; 3 MBBS, MMed Fam Med, Department of Primary Care Medicine, Faculty of Medicine Universiti Teknologi MARA, Jalan Hospital, Sungai Buloh, Selangor, Malaysia.; 4 B. Med. (Sc), MD, M.Community, Health (Epidemiology & Biostat) Department of Public Health Medicine, Faculty of Medicine Universiti Teknologi MARA, Jalan Hospital, Sungai Buloh, Selangor, Malaysia.

**Keywords:** Hyperuricaemia, Primary care, Knowledge, Attitude, Practice

## Abstract

**Introduction:**

The prevalence of hyperuricaemia is increasing worldwide. Hyperuricaemia is associated with many comorbidities, but care quality is suboptimal. This study aimed to determine the level of knowledge, attitude (perceived barrier) and practice in managing asymptomatic hyperuricaemia (AH) among primary care doctors (PCDs) and whether there is a significant difference between PCDs with and without postgraduate qualifications in Malaysia. It also aimed to determine the factors associated with adequate practice in managing AH.

**Methods:**

A cross-sectional study was conducted using a validated online questionnaire. The adequacy of knowledge and practice was set at a score of >50%, and attitude (perceived barrier) was described in percentages. Multiple logistic regression examined the factors associated with adequate practice in managing AH.

**Results:**

A total of 412 PCDs participated, with the majority being women (76.2%) and Malay (74.0%) and working in public primary care clinics (84.0%). The overall mean knowledge score was 24.78 (standard deviation [SD] =3.01), and 96.4% achieved adequacy. For attitude, the most commonly perceived barriers were lack of knowledge about the disease (50%) and guidelines (48.5%). The overall mean practice score was 17.51 (SD=8.09), with 53.2% demonstrating adequate practice. Multivariate analysis identified prior rheumatology attachment as the only significant predictor of adequate practice in managing AH (aOR=1.778, 95% confidence interval=1.083-2.920; P<0.005).

**Conclusion:**

Despite high knowledge levels, a substantial proportion of PCDs report inadequate understanding of AH and its guidelines. Addressing these barriers through targeted educational interventions, guideline dissemination and specialised training, including rheumatology exposure, may enhance AH management in primary care.

## Introduction

Uric acid is the final product of purine and protein metabolism. Uric acid homoeostasis depends on the balance between its production and reabsorption or excretion by the kidneys and intestines.^1^ In about 90% of people with hyperuricaemia, there is either an overproduction of urate, an insufficient urate excretion in the kidneys or both.^2,3^ In addition, an increase in endogenous purine production and the consumption of a high-purine diet can contribute to hyperuricaemia.^2^

The Malaysian Clinical Practice Guideline (CPG) for the Management of Gout (Second Edition) defines hyperuricaemia as a serum urate concentration of >6.8 mg/dL (408 μmol/L).^4^

Asymptomatic hyperuricaemia (AH) is defined as hyperuricaemia without prior gout flares or subcutaneous tophi.^5^

Epidemiological studies have demonstrated a global rise in the prevalence of hyperuricaemia. In Asia, the prevalence of hyperuricaemia is reported mainly in India and China, with rates of 6.4%^6^ and 25.8%,^7^ respectively. These phenomena can be associated with the increasing prevalence of metabolic disorders, overweight and obesity, as well as the consumption of purine-rich food, fructose-sweetened drinks and alcohol.^8,9^ Despite systematic literature searches, there are no data on the prevalence of hyperuricaemia in Malaysia.

Multiple studies have suggested that a diagnosis of hyperuricaemia contributes to the development of other comorbid conditions such as hypertension, chronic renal disease, coronary artery disease and diabetes.^10^ A meta-analysis concluded that hyperuricaemia increases the risk of incident hypertension.^11^ A prospective study showed that hyperuricaemia was likely independently associated with an increased risk of type 2 diabetes mellitus.^12^ Emerging evidence also shows that hyperuricaemia is associated with pre-diabetes.^13^ In addition, hyperuricaemia is associated with metabolic syndrome,^14^ chronic kidney disease and nephrolithiasis.^15^ A systematic review and meta-analysis of 26 studies showed that hyperuricaemia increased the risk of coronary heart disease events.^16^

Many guidelines are available for providing the best clinical practice advice and recommendations for managing hyperuricaemia.^17^ These guidelines, including the Malaysian CPG, recommend against initiating urate-lowering therapy (ULT) for patients with AH in any clinical condition.^5,18^ However, studies have shown that there is a high rate of ULT misuse, suggesting improper prescribing practice due to non-adherence to the guideline.^3,19,20^ This raises a concern about the potentially fatal side effects of ULT, as allopurinol is the leading cause of toxic epidermal necrolysis and Stevens-Johnson syndrome in many countries,^21^ including the local setting in Malaysia.^22, 23^

Gout and hyperuricaemia are regarded as curable illnesses.^24^ However, the present condition is concerning, as studies on knowledge, attitude and practice in the management of AH have revealed that primary care physicians have an insufficient level of essential understanding.^19,25^ Only two published studies worldwide have evaluated the knowledge, attitude and practice in managing AH.^19,25^ To date, there is no published study regarding knowledge, attitude and practice in the management of AH among primary care doctors (PCDs) in Malaysia.

PCDs in Malaysia are referred to as medical officers (MOs) when working in a public clinic and as general practitioners when practising in a private clinic. PCDs with postgraduate training in family medicine are identified as family medicine specialists (FMSs).

This study had several aims. First, this study aimed to determine the level of knowledge, attitude (perceived barrier) and practice in managing AH among PCDs in Malaysia.

Second, it aimed to determine whether there is a significant difference in the level of knowledge, attitude (perceived barrier) and practice in managing AH between PCDs with and without postgraduate qualifications in Malaysia. Finally, this study aimed to test the hypothesis that there is an association of sociodemographic characteristics, level of knowledge and attitude (perceived barrier) with adequate practice in managing AH among PCDs in Malaysia.

## Methods

This cross-sectional study was conducted in two phases from March to September 2022.

### Phase 1

This study adopted the questionnaire by Alqarni to assess the knowledge, attitude and practice in managing AH.^19^ The questionnaire was brief, validated and reliable, with a good Cronbach’s alpha of 0.837-0.972. It was developed in English to assess PCDs in Saudi Arabia. There has been no report of its utilisation in other populations. Thus, a pilot test was conducted to determine its feasibility and reliability among local PCDs. The permission to use this questionnaire was obtained from the developers. The questionnaire was converted into a Google Form to facilitate its accessibility in an online format.

The pilot test had a cross-sectional design and was conducted on 30 PCDs from the Universiti Teknologi MARA Primary Care Clinic, including both FMSs and MOs. The pilot test showed that the online questionnaire was user-friendly and took an average of 15-20 min to complete. There were no cases of missing values. Cronbach’s alpha analysis revealed strong internal consistency for the knowledge (α=0.756), attitude (perceived barrier) (α=0.870) and practice domains (α=0.843).

### Phase 2

This cross-sectional study was conducted using an online questionnaire. PCDs without a postgraduation qualification in family medicine (PCD-noPG-Qual) were defined as those who only had a basic medical degree. PCDs with a postgraduation qualification (PCD-PG-Qual) were those who had completed a postgraduate programme in primary care, such as a Diploma in Family Medicine (DFM), Graduate Certificate of Family Medicine (GCFM), Advanced Training in Family Medicine (ATFM), Master’s in Family Medicine, Fellow of the Royal Australian College of General Practitioners (FRACGP) and Member of the Royal College of General Practitioners (MRCGP), United Kingdom (UK).

The study population was PCDs who worked in a public, private or university primary care clinic. They were invited to participate via email and/or social media messages sent by the three main PCD associations in Malaysia: the Malaysian Family Medicine Specialists’ Association, the Academy of Family Physicians Malaysia and the Malaysian Primary Care Network. Only those who fulfilled the inclusion criteria and consented to the study were included. The inclusion criteria were PCDs who were fully registered with the Malaysian Medical Council with at least 1 year of working experience in a primary care setting in Malaysia and consented to participate in this study. PCDs who participated in phase 1 of this study and were working in primary care clinics as locums only were excluded.

Participants were selected using the convenience sampling method. Reminders were sent 2, 4 and 8 weeks after the initial invitation.

### Study tool

The study questionnaire contained items on sociodemographic characteristics and professional background such as age, sex, specialty, years of practice, average number of patients seen per day and personal experience in managing hyperuricaemia and gout or attachment in rheumatology rotation.

The knowledge domain has 40 items divided into three subdomains: pathophysiology (12 items), common aetiological factors (13 items) and dietary recommendations for people with AH (15 items). For the scoring system, every correct answer scores 1, and every incorrect answer scores 0. Thus, the score ranges from 0 to 40. A knowledge score of ≥20/40 is considered to indicate adequate knowledge (50% of the maximum possible score). The score threshold was chosen based on Alqarni’s conventions for a similar study among healthcare professionals, representing the minimum acceptable level of knowledge rather than expert-level proficiency.^19^

Attitude in managing AH was assessed as perceived barriers (six items). The responses were analysed and presented as frequencies and percentages. The practice domain has 35 items divided into five subdomains: history-taking (eight items), physical examination (four items), evaluation of comorbid conditions (nine items), complementary investigations (eight items) and treatment/management of AH (seven items). The score is calculated and ranges from 0 to 35. A practice score of ≥17.5/35 is deemed to indicate adequate practice (50% of the maximum possible score). This cut-off was also aligned with a prior study to maintain consistency and comparability.^19^

### Sample size

A few sample sizes were calculated based on different prevalence rates according to the aims. The largest sample size was taken according to previous literature, which showed that 49.1% of PCDs were satisfied with their approach to care for patients with AH.^25^ The confidence interval (CI) was set at 95% and the study power at 80%; the minimum sample size required was 384 participants.

### Data entry and statistical analysis

Data entry and statistical analysis were performed using the IBM SPSS Statistics for Windows version 28 (SPSS Inc., Chicago, IL). Descriptive statistics were used to describe the sociodemographic characteristics, professional background and personal experience of participants. Normality testing was conducted for continuous variables using the Kolmogorov-Smirnov test. Continuous data with a normal distribution were presented as means (± standard deviations [SDs]), while non-normally distributed data were presented as medians and interquartile ranges (IQRs). Frequencies and percentages were used to describe categorical data.

The knowledge, attitude (perceived barrier) and practice in managing AH among PCDs were described using descriptive statistics. The attitude (perceived barrier) domain responses were analysed and presented as frequencies and percentages. A chi-square test was conducted to compare the level of knowledge, attitude (perceived barrier) and practice between PCDs with and without postgraduate qualifications. P<0.05 was considered to indicate statistical significance.

The association of the sociodemographic characteristics, knowledge and attitude (perceived barrier) with adequate practice in AH management was examined using inferential analysis. Chi-square and t-tests were conducted to screen factors with P<0.25. These factors were analysed to control for any confounding factors using multiple logistic regression. Statistical significance was set at P<0.05.

## Results

A total of 440 PCDs responded to the online questionnaire (calculated response rate=5%). After data cleaning, 412 PCDs were included, of whom 60.2% (n=248) had no postgraduate qualifications, while 39.8% (n=164) had postgraduate qualifications. The majority were women (76.2%), with no significant sex difference between the groups (P=0.635). The median age was 34.0 (IQR=4) years. The PCD-PG-Qual group was significantly older, with a mean age of 38.4±6.2 years compared to 34.2±4.9 years in the PCD-noPG-Qual group (P<0.001). Ethnic distribution also differed significantly (P<0.001), with a larger proportion of Malay doctors in the PCD-noPG-Qual group. The years of experience in primary care were significantly associated with postgraduate status (P<0.001); most doctors with less than 5 years of experience had no postgraduate qualifications, whereas those with more than 10 years of experience predominantly had postgraduate qualifications. The PCD-PG-Qual group had obtained qualifications, including DFM/GCFM (11.4%), ATFM (4.6%), FRACGP/MRCGP (6.6%) and MMed (Family Medicine) (17.2%). Most PCDs practised in government clinics (84.0%), with no significant differences in the practice setting between the groups (P=0.233). The PCD-PG-Qual group reported seeing significantly fewer patients per day (median=20, IQR=15) compared to the PCD-noPG-Qual group (median=30, IQR=20; P<0.001). Additionally, the PCD-PG-Qual group saw a larger number of patients with gout per month (P=0.006), although the overall number of patients with gout or on ULT did not differ significantly between the groups (P=0.075).

### Awareness of AH

Sixteen PCDs (3.9%) reported that they had never heard of the term ‘AH’, and all of them had no postgraduate qualifications. The difference between the two groups was significant (P<0.001). The majority (84.7%) agreed that gout and AH are different correlated diagnoses. The difference was also significant (P=0.02) (Table 1).

**Table 1:** Awareness of AH among the PCDs (N=412).

Awareness	Total (N=412)	Postgraduate qualification		P-value^a^
		PCD-noPG-Qual group (n=248)	PCD-PG-Qual group (n=164)	
**Whether the PCDs had ever heard of AH**				**<0.001**
Yes	396 (96.1)	232 (58.6)	164 (41.4)	
No	16 (3.9)	16 (100.0)	0 (0.0)	
**PCDs’ opinion on whether AH and gout diagnoses are correlated**				**0.021**
The same diagnosis	9 (2.2)	8 (88.9)	1 (11.1)	
Different correlated diagnosis	349 (84.7)	203 (58.2)	146 (41.8)	
Different uncorrelated diagnosis	45 (10.9)	28 (62.2)	17 (37.8)	
I do not know	9 (2.2)	9 (100.0)	0 (0.0)	

* PCDs: primary care doctors

a Chi-square test

Emboldened: Statistically significant at P<0.05 PCD-noPG-Qual: PCDs without postgraduate qualifications PCD-PG-Qual: PCDs with postgraduate qualifications

### Level of knowledge and practice in managing AH

Table 2 shows that the overall mean knowledge score of all PCDs was 24.78 (SD±3.01), with 96.4% demonstrating adequate practice. The overall mean knowledge score was slightly higher in the PCD-PG-Qual group (24.95±2.80) than in the PCD-noPG-Qual group (24.66±3.15). However, this difference was not statistically significant (P=0.34). In each knowledge subdomain, the PCD-PG-Qual group scored higher than the PCD-noPG-Qual group, but the differences were not statistically significant (P=0.16–0.98)

**Table 2:** Level of knowledge and practice in managing patients with asymptomatic hyperuricaemia among the PCDs (N=412).

Domain	Subdomain	No. of items	Mean (±SD) score of the total PCDs (N=412)	Mean (±SD) score of the PCD noPG-Qual group (n=248)	Mean (±SD) score of the PCD-PG-Qual group (n=164)	Mean score difference (95% CI)	P-value^a^	Cut-off	Adequacy total (N=412)
**Knowledge**									
	Pathophysiology	12	9.01 (±1.50)	9.01 (±1.56)	9.01 (±1.42)	-0.004 (-0.302 to 0.294)	0.978	6.0	402 (97.6)
	Factors/mechanisms	13	4.73 (±1.77)	4.71 (±1.78)	4.75 (±1.76)	-0.036 (-0.387 to 0.315)	0.839	6.5	72 (17.5)
	Diet recommendations	15	11.04 (±1.78)	10.94 (±1.84)	11.19 (±1.68)	-0.250 (-0.601 to 0.102)	0.164	7.5	402 (97.6)
	**Overall knowledge**	40	24.78 (±3.01)	24.66 (±3.15)	24.95 (±2.80)	-0.290 (-0.886 to 0.306)	0.339	20.0	397 (96.4)
**Practice**									
	History-taking	8	3.90 (±3.25)	3.86 (±3.26)	3.96 (±3.24)	-0.094 (-0.738 to 0.549)	0.773	4.0	233 (56.6)
	Physical examination	4	1.94 (±1.77)	1.93 (±1.78)	1.97 (±1.75)	-0.042 (-0.392 to 0.307)	0.813	2.0	223 (54.1)
	Comorbid evaluation	9	3.90 (±3.51)	3.79 (±3.52)	4.06 (±3.51)	-0.271 (-0.966 to 0.425)	0.445	4.5	205 (49.8)
	Complementary investigation	8	4.29 (±2.26)	4.06 (±2.37)	4.65 (±2.04)	-0.596 (-1.040 to -0.152)	**0.009**	4.0	261 (63.3)
	Treatment	6	3.47 (±1.91)	3.26 (±1.90)	3.79 (±1.90)	-0.535 (-0.910 to -0.159)	**0.005**	3.0	261 (63.3)
	**Overall practice**	35	17.51 (±8.09)	16.90 (±8.09)	18.43 (±8.02)	-1.538 (-3.133 to 0.570)	0.059	17.5	219 (53.2)

a T-test

Emboldened: Statistically significant at P<0.05 PCD-noPG-Qual: PCDs without postgraduate qualifications PCD-PG-Qual: PCDs with postgraduate qualifications

The overall mean practice score among all PCDs was 17.51, with 53.2% demonstrating adequate practice. The PCD-PG-Qual group showed a higher overall mean score of 18.43 (SD±8.02) than the PCD-noPG-Qual group (16.90±8.09); however, the difference was not statistically significant (P=0.06). The mean score was higher in the PCD-PG-Qual group in each practice subdomain. However, only the complementary investigation and treatment subdomains significantly differed between the two groups (P=0.009 and P=0.005, respectively). About half of the PCDs achieved adequate performance in history-taking, physical examination, complementary investigation and management (54.1 %–63.3%) but not in comorbidity evaluation (49.8%).

About the prescribing options patterns in managing AH, the majority (98.3%) would recommend a low-purine diet and lifestyle changes to patients with newly diagnosed AH. Conversely, 26.2% and 20.6% would prescribe long-term and short-term ULT to patients with newly diagnosed AH, respectively. Other than that, 18.9% prescribe colchicine and 16.0% prescribe non-steroidal anti-inflammatory drugs (NSAIDs).

### Attitude (perceived barrier) in managing AH

Table 3 shows that inadequate knowledge about the disease (50%) was the most frequently reported perceived barrier in managing patients with AH, followed by a lack of knowledge about the guideline (48.5%) and unawareness of the guideline’s existence (41.5%). The highest percentage was seen in the PCD-noPG-Qual group for these three attitudes (perceived barriers) in AH management. Statistically significant differences were seen in the inadequate knowledge about the disease (P<0.001), unawareness about the existence of guidelines (P=0.025) and lack of knowledge about the guidelines domains (P=0.012) between the PCD-PG-Qual and PCD-noPG-Qual groups. Most of the participants denied a lack of interest in the subject as a barrier to practice adequacy in managing AH.

**Table 3:** Attitude (perceived barriers) in managing AH among the PCDs (N=412).

	Total PCDs (N=412)	Postgraduate qualification		P-value^a^
PCD-noPG-Qual group (n=248)		PCD-PG-Qual group (n=164)		
**Inadequate knowledge about the disease**				
False	36 (8.7)	11 (30.6)	25 (69.4)	**<0.001**
Somewhat true	170 (41.3)	109 (64.1)	61 (35.9)	
True	206 (50.0)	128 (62.1)	78 (37.9)	
**Unawareness about the existence of guidelines**				
False	95 (23.1)	46 (48.4)	49 (51.6)	**0.025**
Somewhat true	146 (35.4)	95 (65.1)	51 (34.9)	
True	171 (41.5)	107 (62.6)	64 (37.4)	
**Lack of knowledge about the guidelines**				
False	45 (10.9)	18 (40.0)	27 (60.0)	0.012
Somewhat true	167 (40.5)	102 (61.1)	65 (38.9)	
True	200 (48.5)	128 (64.0)	72 (36.0)	
**Lack of time**				
False	100 (24.3)	53 (53.0)	47 (47.0)	0.239
Somewhat true	188 (45.6)	118 (62.8)	70 (37.2)	
True	124 (30.1)	77 (62.1)	47 (37.9)	
**Lack of interest in the subject**				
False	181 (43.9)	111 (61.3)	70 (38.7)	0.441
Somewhat true	153 (37.1)	95 (62.1)	58 (37.9)	
True	78 (18.9)	42 (53.8)	36 (46.2)	
**Lack of confidence**				
False	146 (35.4)	90 (61.6)	56 (38.4)	0.428
Somewhat true	174 (42.2)	108 (62.1)	66 (37.9)	
True	92 (22.3)	50 (54.3)	42 (45.7)	

a Chi-square test

Emboldened: Statistically significant at P<0.05 PCD-noPG-Qual: PCDs without postgraduate qualifications PCD-PG-Qual: PCDs with postgraduate qualifications

### Factors associated with adequate practice

Table 4 presents the factors associated with adequate practice in managing AH. Adequate practice was associated with the mean age of the PCDs (P=0.19) but not with sex, ethnicity and duration of practice in primary care. Educational level was also related to adequate practice, in which a significant difference was seen between the PCD-PG-Qual and PCD-noPG-Qual groups (P=0.17). Another factor associated with adequate practice was awareness, which included both items of whether the PCDs had ever heard of the term ‘AH’ (P=0.20) and whether they considered AH and gout to represent the same or a correlated diagnosis (P=0.04). The analysis of the knowledge score showed a significant association between adequate practice and knowledge about pathophysiology (P=0.11). However, there was no association with the overall knowledge score or the other knowledge subdomain scores. Personal experience was also associated with adequate practice, in which the PCDs who had an elective rotation in rheumatology (P=0.02), attended a conference/meeting in rheumatology (P=0.05) and a close relative with hyperuricaemia or gout (P=0.113) reported having more adequate practice. These factors were then included in the multiple logistic regression analysis.

**Table 4:** Factors associated with adequate practice in managing AH among the PCDs (N=412).

Parameter	Practice adequacy		P-value
	Adequate (≥50%) n=219	Inadequate (<50%) n=193	
**Sex**			
Female	162 (51.6)	152 (48.4)	0.255^a^
Male	57 (58.2)	41 (41.8)	
**Mean age (year)**	35.5 (±5.0)	36.3 (±6.7)	0.194^b^
**Ethnicity**			
Malay	166 (54.4)	139 (45.6)	0.572^a^
Chinese	33 (54.1)	28 (45.9)	
Indian	14 (42.4)	19 (57.6)	
Others	6 (46.2)	7 (53.8)	
**Years of practice in primary care**			
0-5	69 (48.6)	73 (51.4)	0.277^a^
6-10	104 (56.8)	79 (43.2)	
11-15	33 (57.9)	24 (42.1)	
>15	13 (43.3)	17 (56.7)	
**Educational level**			
PCD-noPG-Qual	125 (50.4)	123 (49.6)	**0.169** ^a^
PCD-PG-Qual	94 (57.3)	70 (42.7)	
**Place of practice**			
Government clinic	188 (54.3)	158 (45.7)	0.446^a^
Private clinic	18 (43.9)	23 (56.1)	
University clinic	13 (52.0)	12 (48.0)	
**Mean number of outpatients seen per day**	29.0 (±16.1)	30.4 (±15.6)	0.371^b^
**Average number of patients with gout seen monthly**			
None/I do not know	3 (37.5)	5 (62.5)	0.395^a^
1-5	137 (54.6)	114 (45.4)	
6-10	63 (54.8)	52 (45.2)	
>10	16 (42.1)	22 (57.9)	
Mean total number of patients with gout or under ULT	15.0 (±15.0)	15.1 (±39.3)	0.954^b^
**Whether the PCD had ever heard of AH**			
Yes	213 (53.8)	183 (46.2)	0.201^a^
No	6 (37.5)	10 (62.5)	
**Opinion on AH and gout representing**			
The same diagnosis	3 (33.3)	6 (66.7)	**0.040** ^a^
Different correlated diagnosis	189 (54.2)	160 (45.8)	
Different uncorrelated diagnosis	26 (57.8)	19 (42.2)	
I do not know	1 (11.1)	8 (88.9)	
**Knowledge – pathophysiology score**			
Adequate (≥6/12)	211 (52.5)	191 (47.5)	**0.112** ^a^
Inadequate (<6)	8 (80.0)	2 (20.0)	
**Knowledge –factor/mechanism score**			
Adequate (≥6.5/13)	34 (47.2)	38 (52.8)	0.267^a^
Inadequate (<6.5)	185 (54.4)	155 (45.6)	
**Knowledge – dietary score**			
Adequate (≥7.5/15)	214 (53.2)	188 (46.8)	0.840^a^
Inadequate (<7.5)	5 (50.0)	5 (50.0)	
**Knowledge – overall score**			
Adequate (≥20/40)	213 (53.7)	184 (46.3)	0.298^a^
Inadequate (<20)	6 (40.0)	9 (60.0)	
**Personal experience**			
**Done elective rotation in rheumatology**			
Yes	54 (64.3)	30 (35.7)	**0.022** ^a^
No	165 (50.3)	163 (49.7)	
**Attended conferences or meetings in rheumatology**			
Yes	92 (59.4)	63 (40.6)	**0.050** ^a^
No	127 (49.4)	130 (50.6)	
**Self-afflicted with HU/gout**			
Yes	39 (57.4)	29 (42.6)	0.448^a^
No	180 (52.3)	164 (47.7)	
**A close relative with HU/gout**			
Yes	93 (48.9)	97 (51.1)	**0.113** ^a^
No	126 (56.8)	96 (43.2)	

a Chi-square test

b T-test

Emboldened: Statistically significant at P<0.25 PCD-noPG-Qual: PCDs without postgraduate qualifications PCD-PG-Qual: PCDs with postgraduate qualifications

### Predictor of adequate practice

The multiple logistic regression analysis identified a single factor significantly associated with adequate practice (Table 5). The PCDs who had an elective rotation in rheumatology were 1.778 times more likely to achieve adequate practice in managing AH (OR=1.778, CI=1.083–2.92; P=0.02).

**Table 5:** Predictor of adequate practice in managing AH.

Characteristic	Adjusted B	Standard error	Wald (df)	Adjusted OR (95% CI)	P-value
**Done elective rotation in rheumatology**					
No	-	-	-	Ref	-
Yes	0.576	0.253	5.173 (1)	1.778 (1.083, 2.920)	0.02

Multiple logistic regression; forward LR method Statistical significance at P<0.05 Ref: reference group

## Discussion

This study showed that the Malaysian PCDs had a high overall level of knowledge on AH. The PCDs were most informed about pathophysiology and dietary recommendations (97.6%) but showed a poor understanding of the factors and mechanisms of AH (17.5%). In comparison with Alqarni’s findings, the level of knowledge was also low for factors and mechanisms (3%) and moderate for pathophysiology (47.3%) and dietary recommendations (62.7%).^19^ Another study conducted in 2021 revealed that Croatian primary care physicians had moderate knowledge of gout and AH management.^25^

As for attitude (perceived barrier), the PCDs without postgraduate qualifications believed that their inadequate knowledge about the disease was the greatest impediment to managing AH. Although the majority of the PCDs in this study had a high overall level of knowledge, this paradoxical result may be reflected by the poor understanding of the factors and mechanisms of AH. The other perceived barriers were a lack of knowledge about the guideline and unawareness of the existence of guidelines. Alqarni’s findings are comparable with our data in that these three attitudes were the most commonly perceived barriers among doctors in Saudi Arabia.^19^ Similarly, only 3.3% of Croatian primary care physicians fully agreed that they were familiar with the European League Against Rheumatism guidelines.^25^ Strategies to enhance the understanding of current guidelines and education on the factors and mechanisms of AH among PCDs are crucial to lower the risk of comorbidities associated with hyperuricaemia.

This study demonstrated a moderate practice adequacy in managing AH among the PCDs in Malaysia. We observed that the PCDs with postgraduate qualifications performed better in the practice subdomains of complementary investigation and treatment. These may indicate that PCDs with postgraduate qualifications have a better comprehension of the guidelines, as they exercise greater caution when performing additional investigations and determining whether to initiate ULT in patients with AH. Complementary investigations may be helpful in diagnosing patients with AH, such as an ultrasound to detect subclinical arthritis^26^ or a urine uric acid test to detect urate overproduction.^27^ However, there are currently no guidelines that advocate these approaches. Patients with urate deposition on imaging are still considered to have AH if they have never experienced a gout flare or subcutaneous tophi.^5^

Some PCDs without postgraduate qualifications were found to have never heard of AH, and others responded that they were unsure about the relationship between gout and AH diagnoses. Hyperuricaemia is not equivalent to gout.^28^ Nevertheless, AH and gout are correlated diagnoses in which hyperuricaemia is a primary risk factor for gout.^29^ The presence of monosodium urate crystals in synovial fluid or tophi is a gold-standard finding for a definite diagnosis of gout, as recommended by all CPGs for gout.^17^ However, the diagnosis of AH is based on ruling out whether the patient has gout symptoms or an undiagnosed gouty attack. Both gout and hyperuricaemia are commonly managed diseases in primary care clinics; hence, it is essential that PCDs receive proper education and training in AH management.

The total number of patients with gout on ULT was found to be lower among the PCDs without postgraduate qualifications. One potential explanation is that all ULT drugs available in Malaysia, such as allopurinol, probenecid and febuxostat, are classified as category A in the Medicine Formulary of the Ministry of Health Malaysia No. 2/2022. This classification restricts the prescription of these medications to consultants or specialists in the relevant specialty only. This may be attributed to apprehension of incorrect prescription, which could lead to severe adverse reactions caused by the medications.

Worldwide guidelines for managing AH recommend that the only therapeutic option is a low-purine diet and lifestyle modifications; the majority of the PCDs in this study concurred with this, as did participants in Alqarni’s study. Dietary and therapeutic lifestyle adjustments are the most important strategies to minimise the risk of comorbidity associated with AH; these include restricting the intake of high-purine foods as well as managing obesity, smoking and alcohol intake.

However, almost half of the PCDs in this study stated that they would prescribe either shortterm or long-term ULT for AH. The number is almost similar to that in Alqarni’s study.^19^ Generally, international guidelines do not recommend starting ULT in the management of AH. In Mustafa’s study, 84.1% of physicians said they would recommend allopurinol treatment for AH.^3^ It is also a common practice by UK physicians to prescribe allopurinol for AH, suggesting improper prescribing practice due to non-adherence to the guideline.^20^ This may raise a concern about the potentially fatal side effects of ULT. Other potentially misprescribed medicines include colchicine (18.9%) and nonsteroidal anti-inflammatory drugs (NSAIDs) (16.0%). These values are higher than those in Alqarni’s study, with colchicine at 1% and NSAIDs at 10.9%.

The final regression analysis revealed that the PCDs who had an elective rheumatology rotation had a greater practice adequacy. The PCDs with postgraduate qualifications most likely acquired this experience since Malaysian training involves a rheumatic clinic rotation. This prompted the question of whether every PCD should have a rheumatology attachment to improve their practice adequacy in managing AH. The collaboration between rheumatology physicians and PCDs in continuous medical education sessions and continuous professional development activities for gout and hyperuricaemia may be beneficial, as the raised question seems unreasonable to achieve.

This is the first study to examine the knowledge, attitude (perceived barrier) and practice in managing patients with AH among PCDs in Malaysia. However, this study also has several limitations. Due to the cross-sectional design of the study, the findings can only establish a correlation, rather than a causal relationship, between the demographic characteristics and knowledge, attitude (perceived barrier) and practice in managing AH. Convenience sampling via an online recruitment approach was selected for its practicality and feasibility; however, this non-probability sampling method may be susceptible to sampling bias. To mitigate this bias, we ensured that the invitation for participation in this study was widely distributed to PCDs through the nation’s three main PCD organisations and multiple internet and social media platforms, with regular reminders. Lastly, other factors could influence the practice in managing AH, which were not investigated, as this is beyond the scope of this study. Thus, the results from the multiple logistic regression should only be interpreted based on the variables contained in the regression model.

## Conclusion

In conclusion, this study revealed important insights into Malaysian PCDs’ knowledge, attitude (perceived barrier) and practice in managing AH. We discovered that despite having an adequate overall knowledge, the PCDs showed a significant gap in their knowledge of the factors and mechanisms that lead to hyperuricaemia. The primary perceived barrier to adequate practice in managing AH was a lack of understanding of the condition, followed by a lack of awareness and knowledge about the guidelines. The PCDs had a moderate practice adequacy in managing AH, with a noticeably high rate of ULT abuse. The PCDs with a rheumatology clinic rotation demonstrated a significantly higher practice adequacy. This implies that PCDs in Malaysia require more comprehensive training in managing AH, particularly in the clinical component.

## Data sharing statement

The datasets collected and analysed during the study are available upon reasonable request from the corresponding author and are subject to data protection laws and regulations.

**How does this paper make a difference in general practice?**

- This study identifies a significant knowledge gap among primary care doctors (PCDs) in understanding and managing asymptomatic hyperuricaemia (AH), particularly regarding the disease itself and existing guidelines.
- The study also reveals that previous clinical attachment with a rheumatology team is significantly associated with better practices in managing AH.
- It also emphasises the need for increased rheumatology exposure through clinical attachments or continuous medical education for PCDs.
- The findings suggest that strengthening collaboration between primary care and rheumatology teams can enhance patient care and ensure alignment with current clinical recommendations.
